# Preoperative and Postoperative Ozone Therapy in Cats Presenting Extensive Wounds Treated by Reconstructive Surgery Methods—A Short Case Series

**DOI:** 10.3390/vetsci12080786

**Published:** 2025-08-21

**Authors:** Nicușor Valentin Oros, Călin Cosmin Repciuc, Lucia Victoria Bel, Iulia Melega, Andreea Niculina Pertea, Liviu Ioan Oana

**Affiliations:** 1 Department of Internal Medicine, Faculty of Veterinary Medicine, University of Agricultural Sciences and Veterinary Medicine, 400372 Cluj-Napoca, Romania; nicusor-valentin.oros@usamvcluj.ro; 2 Department of Surgery Anesthesiology and Intensive Care, Faculty of Veterinary Medicine, University of Agricultural Sciences and Veterinary Medicine, 400372 Cluj-Napoca, Romania; lucia.bel@usamvcluj.ro (L.V.B.); iulia.melega@usamvcluj.ro (I.M.); andreea-niculina.astilean@usamvcluj.ro (A.N.P.); liviu.oana@usamvcluj.ro (L.I.O.)

**Keywords:** cat, ozone, skin reconstructive surgery, wounds, complementary therapy

## Abstract

The therapeutic management of extensive skin wounds in cats can be time-consuming and require multiple therapeutic interventions, which can have significant financial implications for pet owners. Reconstructive surgery is often necessary to close skin defects with tissue loss to provide a quicker patient recovery. Conventional therapies like systemic antibiotics, anti-inflammatories, and local dressings are not always successful due to antibiotic resistance or a poor response, such as no or delayed healing. For more than a century, ozone has been utilized as an excellent disinfectant, but caution should be taken due to its oxidizing properties. Only in the past decade have numerous studies established therapeutic dose ranges for a wider medical use of ozone. The objective of this study was to clinically evaluate ozone therapy as a complementary treatment supporting and completing plastic and reconstructive surgery in 4 cats with extensive skin defects. The results obtained, following the local application of ozone therapy before and after skin reconstruction in our patients, encourage the use of ozone as a complementary therapy in the management of extensive skin wounds treated surgically by different reconstructive techniques.

## Simple Summary

Wound therapy in cats is often challenging for the veterinary practitioner. The positive therapeutic effect of ozone therapy has been well documented and results from the chemical reaction with certain tissue constituents. We present here the clinical evolution of four feline patients being treated with both reconstructive surgical procedures and ozone therapy as a complementary therapeutic method to support the preoperative surgical preparation and the postoperative healing process. Based on the results, the authors recommend integrating this therapy into the management of feline skin wounds.

## 1. Introduction

Extensive wounds in cats are a common encounter in current practice, and their healing is closely related to the level of microbial contamination, the size and location of the wound, associated pathologies, and the amount of tissue loss. The evaluation, management, and choice of the therapeutic methods must be thought out as well as possible to obtain positive and quick results [1]. To restore homeostasis to the affected area, it is ideal to use therapies that can improve wound healing by increasing blood supply, controlling the local infectious process, and releasing the growth factors necessary for the healing process [2].

Medical ozone is a colorless gas with a specific odor formed by the union of three oxygen atoms. The concentration range of medical ozone is between 0.5 and 5%, corresponding to a range of 1–80 μg/mL (microgram of ozone/milliliter of oxygen), obtained from medical oxygen, using medical generators [3,4].

Ozone therapy is a complementary method established decades ago, but it still encounters some resistance within the medical community. Although ozone (O_3_) can have dangerous effects in high concentrations, researchers have noted that when used at appropriate doses, it has therapeutic benefits. Thus, ozone therapy and its multiple routes of administration and effects on the human and animal body have been thoroughly studied for more than a century worldwide.

Local ozone therapy used as a complementary method in wound management supports healing by improving capillary circulation, increasing oxygenation levels, modulating the immune system, and having antibacterial potential [5,6]. The scarring phase is improved, due to the positive impact of ozone on angiogenesis, fibroblast activity, and collagen production [7,8,9].

The therapeutic efficacy of ozone is partly due to oxidative stress, which is controlled and moderated by ozone reactions with biological fluids and body tissues. The boundary between efficacy and toxicity in ozone therapy is given by the level of oxidative stress induced by it [10]. Following ozone exposure of tissues, reactive oxygen species (ROS) and lipid oxidation products (LOP) are formed. Reactive oxygen species act rapidly, following a transient decrease in antioxidant capacity. In this context, hydrogen peroxide is the most important molecule in the group of reactive oxygen species, being a non-radical oxidant, which plays the role of a signaling molecule in the intracellular environment, being a messenger of the therapeutic dose of ozone [11]. Through the cell membrane and cytosol, hydrogen peroxide activates the erythroid factor 2 nuclear pathway (Nrf2) and protein synthesis, which promotes cell survival. In controlled doses, ozone accelerates the cell cycle and induces growth factor synthesis by activating redox transcription factors such as nuclear factor kappa B (NFkB) [8]. NFkB is an activator for interleukin 8 (IL-8), tumor necrosis factor α (TNF-α), transforming growth factor beta (TGF-β), and is a regulator for inflammatory responses [9]. A possible decrease in the rate and maturation of collagen production, as well as slower development of granulation tissue in the cat compared to the dog [12], could benefit from the use of complementary therapies to improve these shortcomings.

Considering the therapeutic properties of ozone, we proposed its clinical evaluation as a healthy granulation tissue preparation factor before grafting and as a support therapy after reconstructive surgery in four cats with extensive skin defects showing a seriously altered general condition.

The objective of this study was to describe the clinical course and evaluate the potential contribution of preoperative and postoperative ozone therapy as an adjunct to reconstructive surgical treatment of extensive skin wounds in four feline patients. This objective is descriptive in nature, and the findings should be interpreted within the limitations of a small, uncontrolled case series.

## 2. Description of Cases

Four cats with extensive skin defects were evaluated at the Surgery Clinic of the Faculty of Veterinary Medicine in Cluj-Napoca. Patient stewards were informed about the ozone therapy and the procedures performed, obtaining their written consent. Each patient benefited from neurological, radiological, hematological, biochemical, and microbiological examination of skin defects. The hospitalization time for each patient was variable, related to the duration of the therapy (hydro electrolyte restoration, analgesics, antibiotics, dressing changes), other special needs, and surgical procedure duration. The rapid test for detecting antibodies against feline immunodeficiency virus and feline leukemia virus antigens (FIV Ab/FeLV Ag, VetExpert, Łomianki, Poland was negative for all patients.

### 2.1. Case 1

**Figure 1:**
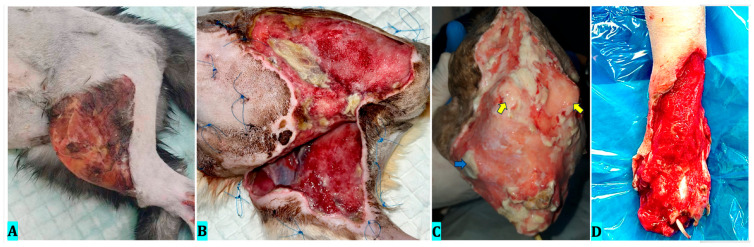
Clinical appearance of wounds before initiating ozone therapy. (**A**)—Case 1 (initial presentation); (**B**)—Case 2 (initial presentation); (**C**)—Case 3 (7 days after the initial presentation—where the blue arrow represented the left hip area and the yellow arrows represented the iliac crest); (**D**)—Case 4 (initial presentation).

An intact 2-year-old female cat presented with a wound showing significant tissue loss at the level of the left hind limb (Figure 1A). The cause of this wound was unknown, most likely of traumatic origin, given that the posterior right limb had a diaphyseal tibial fracture. Hematological examination revealed anemia (RBC 5.42 × 10^12^/L; 7.7 to 12.8^12^/L, HGB 7.1 g/dL; 10.0 to 17.0 g/dL, HCT 22.21%; 33.7 to 55.40%). After debridement of the devitalized tissues covering the wound and its edges, a defect of 96.7 cm^2^ resulted, and ozone therapy was initiated on the same day. Microbiological examination revealed *Staphylococcus* spp. bacteria, susceptibility to marbofloxacin and amikacin. Marbofloxacin 4 mg/kg was used subcutaneously for 7 days (Marfloxin 20 mg/mL, KRKA, Novo Mesto, Slovenia). Three days after the presentation, the tibia fracture was repaired. Analgesic protocol during the wound healing was represented by buprenorphine 0.02 mg/kg, intravenously (Bupaq 0.3 mg/mL, Richter Pharma, Wels, Austria) at 12 h, for 5 days, and robenacoxib 1 mg/kg, orally (Onsior 6 mg, Elanco GmbH, Bad Homburg vor der Höhe, Germany), once daily, for the next 5 days. After reconstructive surgery, the patient received buprenorphine 0.02 mg/kg, intravenously (Bupaq 0.3 mg/mL, Richter Pharma, Wels, Austria) at 12 h, for 3 days.

### 2.2. Case 2

A female cat, a common breed, 5 years old, was brought in critical condition with a post-amputation dehiscent wound hind limb (Figure 1B). The patient had a considerable skin defect in the left flank and sacral region, as well as in the groin and medial area of the right hind limb. Hematologic examination revealed severe anemia without signs of regeneration (RBC 3.29 × 10^12^/L; 5 to 10^12^/L, HGB 4.5 g/dL; 8 to 17 g/dL, HCT 14.38%; 24 to 45%), thrombocytopenia (PLT 130 × 10^9^/L; 300 to 800 × 10^9^/L). Biochemistry revealed hypoalbuminemia (1.06 g/dL; 1.92 to 3.3 g/dL). Hemodynamic, electrolyte, and analgesic stabilization procedures were performed. The wound was subsequently debrided, resulting in a defect measuring 163.2 cm^2^. Ozone therapy was started on the same day, after debridement. Bacteriological examination revealed *Pseudomonas* spp., susceptibility to amikacin. Amikacin was used for 10 days at a dose of 10 mg/kg intravenously once daily (Amikozit 500 mg/2 mL, Zentiva, Bucharest, Romania) and metronidazole for 7 days at a dose of 15 mg/kg intravenously at 12 h (Metronidazole Braun 5 mg/mL, B. Braun, Melsungen AG, Germany). Analgesic protocol during the wound healing was represented by buprenorphine 0.02 mg/kg, intravenously (Bupaq 0.3 mg/mL, Richter Pharma, Wels, Austria) at 8 h, for 5 days, and meloxicam 0.1 mg/kg, orally (Meloxoral 0.5 mg/mL, Oudewater, The Netherlands), once daily first administration, and 0.05 mg/kg for the next 10 days. After reconstructive surgery, the patient received methadone 0.3 mg/kg, intramuscularly (Insistor 10 mg/mL, Richter Pharma, Wels, Austria) at 6 h, for 3 days, and meloxicam 0.05 mg/kg, orally for 10 days.

### 2.3. Case 3

A female cat, found and brought to the veterinary hospital, presented in critical condition. Unstable patient, nonambulatory, altered state of consciousness, hypothermic 32 °C, hypotensive (SP 65 mmHg) with an open tibial diaphyseal fracture in the right limb, a compromised distal extremity of the same limb, tail avulsion, an extensive tissue necrosis in the lumbosacral area, and cutaneous myiasis. Haematological examination revealed severe anemia (RBC 3.06 × 10^12^/L; 7.7 to12.8^12^/L, HGB 4.1 g/dL; 10.0 to 17.0 g/dL, HCT 13.82%; 33.7 to 55.40%) PLT thrombocytopenia (8 × 10^9^/L; 125 to 618 × 10^9^/L) leukopenia (WBC 0.83 × 10^9^/l; 3.50 to 20.709/L) and neutropenia (NEU 0.52 × 10^9^/L; 1.63 to 13.379/L). Biochemistry revealed hypoproteinemia (ALB 1.1 g/dL; 2.4 to 3.7 g/dL, TP 3.4 g/dL; 5.7 to 8.0 g/dL, GLOB 2.3 g/dL; 2.4 to 4.7 g/dL). After performing stabilization procedures that required the correction of hydro-electrolytic imbalances, acid-base and hematological abnormalities, and administration of analgesic medication, the right hind limb was amputated using a midfemoral technique, and lumbosacral necrotic tissue was removed. Ozone therapy was started 7 days after the initial presentation of the patient, the wound having an area of 64.8 cm^2^ at the moment (Figure 1C). Bacteriological examination revealed mixed bacterial flora, including G^–^, *Escherichia coli*, *Proteus* spp., and *Pseudomonas aeruginosa*, with susceptibility to gentamicin. Gentamicin was used for 10 days at a dose of 5 mg/kg intravenously once daily (Gentamicin inj., 50 mg/mL, VETERIN SA, Atena, Greece) and metronidazole for 10 days at a dose of 15 mg/kg intravenously at 12 h (Metronidazole Braun 5 mg/mL, B. Braun, Melsungen AG, Germany). Analgesic protocol for the first 7 days was represented by fentanyl CRI 0.01 mg/kg/h (Fentanyl Torrex 0.05 mg/mL, Vienna, Austria). For the next 15 days, this patient received meloxicam 0.1 mg/kg, orally (Meloxoral 0.5 mg/mL, Oudewater, The Netherlands), once daily, first administration, and 0.05 mg/kg for the next administration.

### 2.4. Case 4

An intact 1.5-year-old male cat, mixed-breed, with an extensive wound at the level of the right forelimb’s dorsal metacarpal-phalangeal area due to a car accident. After surgical debridement of the wound, its surface area was 34 cm^2^, affecting approximately 80% of the limb diameter (Figure 1D). Ozone therapy was started on the same day, after debridement. The bacteriological examination was negative. The patient did not receive antimicrobial therapy. Analgesic protocol for the first 5 days was represented by buprenorphine 0.02 mg/kg, intravenously (Bupaq 0.3 mg/mL, Richter Pharma, Wels, Austria) at 8 h and meloxicam 0.1 mg/kg, orally, for the first administration, and 0.05 mg/kg for the next 11 administrations. After a mesh skin graft was performed, the patient received robenacoxib 1 mg/kg, orally (Onsior 6 mg, Elanco GmbH, Bad Homburg vorder Höhe, Germany), once daily, for 5 days.

The patients did not receive antibiotic treatment after the surgical procedures and benefited only from analgesic protocols and ozone therapy to maintain the microbial clearance of the grafts and their grafting sites.

## 3. Ozone Therapy

All patients after stabilization benefited from the debridement of non-viable tissues, and then the surface of the defect was measured using a thin plastic foil, tracing on it the edges of the skin lesion, followed by transcription on graph paper to obtain the total area. This study did not include a control group, and all patients received concurrent antimicrobial therapy before the surgical interventions. As such, it was not possible to isolate the independent effect of ozone therapy from these other therapeutic methods. The role of ozone was therefore evaluated descriptively, focusing on its use in wound bed preparation before reconstructive surgery and in the postoperative period to maintain adequate wound condition.

**Figure 2:**
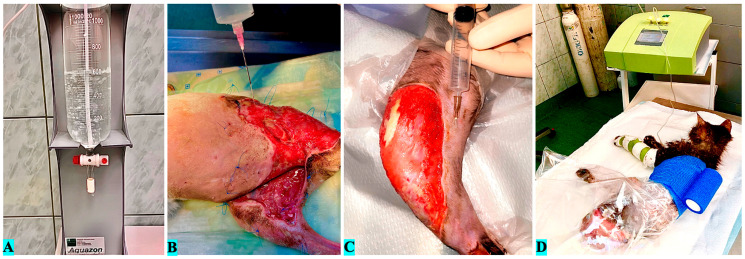
Methods of local application of ozone therapy. (**A**)—Ozone bubbling of saline solution (**B**)—Lavage with ozonized solution, (**C**)—Perilesional subcutaneous infiltration method, (**D**)—Plastic bagging method.

The ozone used to perform the therapy was produced by a medical generator (Medozon Compact; Herrmann Apparatebau GmbH, Elsenfeld, Germany) from medical oxygen. Medical extensions for intravenous fluid administration, polyethylene bags, Vetrap, 2 mL, 20 mL, and 50 mL syringes, 18 G and 26 G dermal needles, and a glass bubbler (Aquazon; Herrmann Apparatebau GmbH, Germany) were used for the therapy. The lavage solution consisted of 0.9% sodium chloride, obtained extemporarily, by bubbling (Figure 2A) for 20 min at a concentration of 60 μg/mL.

While ozone concentration, exposure time, and frequency were adapted to wound characteristics and stage of healing, a general protocol was followed: (1) preoperative phase—concentrations of 40–60 μg/mL for antibacterial effect via bagging (Figure 2D), combined with ozonized saline lavages (Figure 2B) and perilesional subcutaneous infiltration (Figure 2C) at 15 μg/mL; (2) transition to regenerative phase—concentrations of 10–30 μg/mL every 3 days for granulation and epithelialization support; (3) postoperative phase—bagging at 20 μg/mL every 3 days until dressings were no longer required. Adjustments were made for wound size, bacterial load, and observed healing progression to ensure safety and practicality.

The administration methods were in line with the Madrid Declaration of 2020 and WFOT’s Review on Evidence-Based Ozone Therapy from 2015 [4,13]. Simple dressings consisting of sterile gauze, orthopedic cotton, gauze cloth, and Vetwrap were used.

Ozone therapy was performed preoperatively, until the wounds had developed healthy granulation tissue, without clinical signs of bacterial infection, to support the cutaneous flap or the skin graft. The granulation tissue was considered optimal when it had a bright red color, was evenly distributed over the wound surface, and had the presence of epithelialization at the wound edges. Post-surgery, the remedied defects benefited from ozone therapy only by bagging, every 3 days, for a variable period depending on the evolution of each patient, at which time the dressings were also changed.

## 4. Surgical Procedure and Results

### 4.1. Case 1

**Figure 3:**
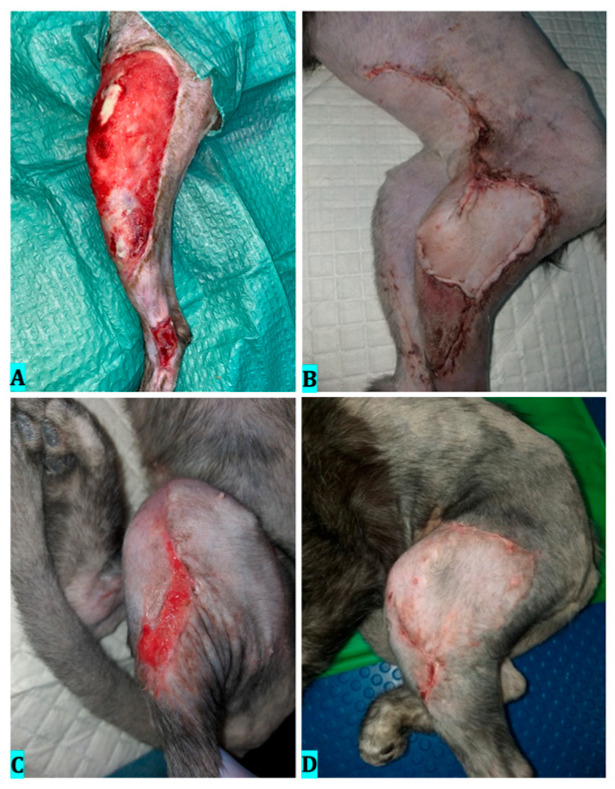
Clinical evolution of wound healing of case 1. (**A**)—Wound on day 15, just before surgery, presenting healthy granulation tissue; (**B**)—Wound appearance 3 days after surgery; (**C**)—Wound 14 days after surgery; (**D**)—60 days after first clinical presentation.

*Per secundam intentionem,* wound management was used for 15 days. Ozone therapy was used before surgery by bagging, for 3 consecutive days, at a concentration of 60 μg/mL, with an exposure time of 5 min, lavage with ozonated saline solution, and ozone subcutaneous infiltrations at a concentration of 15 μg/mL. The following sessions were performed every 3 days by bagging at a concentration of 30 μg/mL, with an exposure time of 20 min and lavage with ozonized saline. On the 15th day after being presented to our hospital, the wound showed healthy granulation tissue with epithelialized edges (Figure 3A). This was an optimal condition to close the defect by an axial flap vascularized by the superficial caudal epigastric artery (Figure 3B). Postoperatively, the patient also benefited from 3 sessions of ozone therapy by bagging, every 3 days, with a concentration of 20 μg/mL and an exposure time of 20 min. In this case, the graft/flap viability was estimated at approximately 95% after 14 days (Figure 3C), defined as the proportion of the wound surface with viable tissue and intact epithelial coverage. After 30 days, the patient had fully recovered (Figure 3D). In total, 10 ozone therapy sessions were performed postoperatively.

### 4.2. Case 2

**Figure 4:**
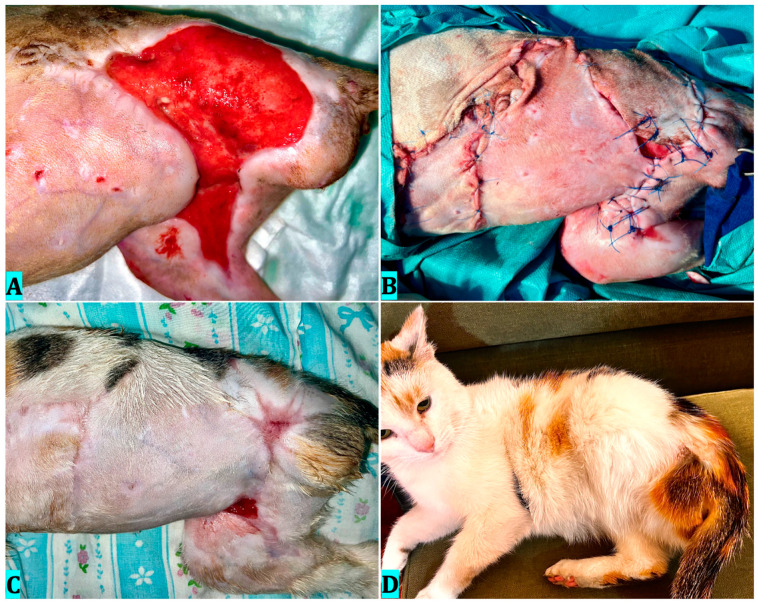
Clinical evolution of case 2. (**A**)—Wound on day 20, before surgery, showing healthy granulation tissue; (**B**)—Wound appearance after surgery; (**C**)—Wound 30 days after surgery; (**D**)—90 days after first clinical presentation.

Ozone therapy was performed daily for the first 5 days by bagging, at a concentration of 60 μg/mL, with an exposure time of 5 min, and by subcutaneous infiltrations, at a concentration of 15 μg/mL, in volumes of 0.5 mL per point of administration, and lavages with ozonated saline. The following five therapies were performed every 3 days by bagging, at a concentration of 30 μg/mL, with an exposure time of 20 min, ozone infiltration at a concentration of 15 μg/mL, and lavage with ozonated saline. On day 20, the wound showed a favorable evolution with optimal granulation tissue development (Figure 4A). The wound edges were adherent to the underlying tissues, undergoing epithelialization, reducing their size by 40%. Considering this, the decision to perform the surgery with the technique of local advancing skin flaps was taken (Figure 4B). Post-operatively, the patient had seven sessions of ozone therapy by bagging, at a concentration of 20 μg/mL, with an exposure time of 20 min every 3 days. The success rate of the skin reconstruction was approximately 90% (Figure 4C); 90 days after the initial presentation, the patient had almost fully recovered (Figure 4D), mentioning that a small area in the groin showed healing deficiency due to increased mobility.

### 4.3. Case 3

**Figure 5:**
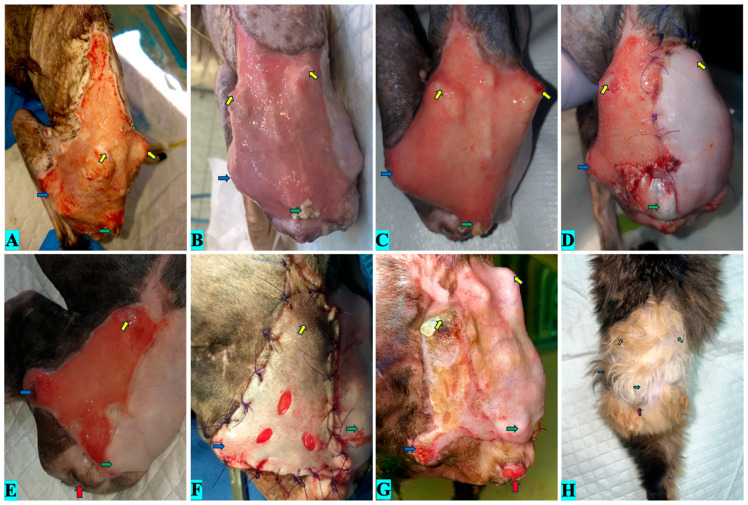
Clinical evolution of case 3. (**A**)—Wound on day 10 of ozone therapy; (**B**)—Wound on day 20 of ozone therapy; (**C**)—Wound on day 30 of ozone therapy (before first surgery); (**D**)—Wound appearance 3 days after first surgery; (**E**)—Wound appearance before second surgery; (**F**)—Wound appearance after mesh skin graft surgery; (**G**)—20 days after graft surgery (day 65); (**H**)—90 days after first clinical presentation (red arrow represented anus, green arrow represented the base of the tail, blue arrow represented hip area and yellow arrows represented iliac crest).

The wound was subjected to daily ozone therapy for the first 10 days (Figure 5A) by bagging, at a concentration of 60 μg/mL for 5 min, subcutaneous infiltrations, at a concentration of 15 μg/mL, in volumes of 0.5 mL per point of administration, and lavages with ozonated saline. For the next 21 days, the therapy was performed every 3 days by bagging, at a concentration of 30 μg/mL for 20 min, ozone infiltrations at a concentration of 15 μg/mL, and lavage with ozonated saline (Figure 5B,C). Subsequently, part of the defect was reduced with skin flaps resulting from the amputation of the right limb by cox-femoral disarticulation (Figure 5D). The remaining wound had a total area of 21.4 cm^2^ (Figure 5E).

A second plastic surgery was performed and consisted of a free skin graft harvested from the left thorax. Before the second surgery, the patient had 3 days of bagging ozone therapy and 15 days of lavages with ozonated saline, helping with the epithelialization of approximately 60% of the defect. After applying the skin graft (Figure 5F), four sessions of bagging were performed at a concentration of 20 μg/mL. After 20 days, the graft showed a viability of approximately 95% (Figure 5G), and no more dressing changes were needed. After 90 days at final follow-up, the wound demonstrated complete epithelial coverage with hair regrowth and no visible contracture or ulceration; cosmetic outcome was subjectively assessed as optimal (Figure 5H).

### 4.4. Case 4

**Figure 6:**
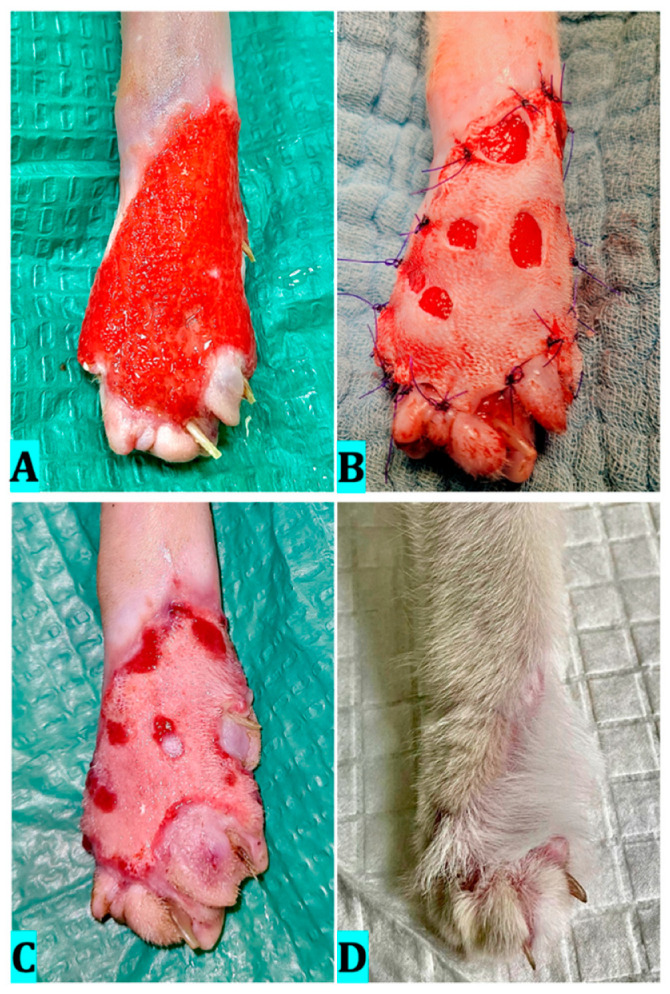
Clinical evolution of case 4. (**A**)—Healthy granulation tissue developed after 20 days with ozone therapy; (**B**)—The skin graft after application; (**C**)—Nine days after grafting; (**D**)—The appearance of the limb 60 days after grafting.

Ozone therapy was performed by bagging for 5 days at a concentration of 60 μg/mL and wound cleansing with ozonated saline. The following sessions were performed every 3 days by 20 min bagging at a concentration of 30 μg/mL and lavages with ozonated saline. On day 20, the wound had developed healthy granulation tissue (Figure 6A), and we decided to perform the skin graft (Figure 6B). Ozone therapy by bagging was performed postoperatively every 3 days, until the 15th day, with a concentration of 20 μg/mL. Graft viability after 9 days (Figure 6C) was 100%, with evident areas of epithelialization. After 15 days of post-surgical therapy, the wound healed completely, and no further dressing application was needed. Hair growth was observed in the grafted area by day 60 postoperatively; however, while histological examination was not performed, the visible return of hair growth suggests a favorable long-term outcome (Figure 6D). A simple dressing consisting of sterile nonadherent gauze and adhesive wrap was used after each ozone therapy session, so as not to influence the results.

The wound epithelialization was scored for each stage presented in the figures, at presentation, after completion of initial preoperative therapy, and the final postoperative stage representing the wound condition at the end of the follow-up period. Scores were based on a standardized 0–5 scale (0 = no epithelial coverage and low granulation, 5 = complete epithelial coverage). Approximate percentage coverage values were estimated from the clinical evaluation.

The initial wound areas ranged from 34 cm^2^ to 163.2 cm^2^. Three patients had confirmed bacterial infections with pathogens including *Staphylococcus* spp., *Pseudomonas* spp., *Escherichia coli*, and *Proteus* spp., while the fourth case had a negative bacteriological examination. All patients underwent a multimodal therapeutic approach involving a preoperative management phase with ozone therapy, which lasted between 15 and 31 days, and a subsequent reconstructive surgical procedure (2 procedures for case 3). The surgical techniques included axial flaps, local advancing flaps, and free skin grafts. The total time to achieve complete healing ranged from 35 to 90 days. The patient with the largest wound (case 2) and the most complex case (case 3) had the longest healing times. This suggests a correlation between the initial wound size, complexity, and overall time required for complete healing as summarized in Table 1.

Photographic evaluation of each case demonstrated progressive epithelialization across all patients from the preoperative stage to final follow-up. Table 2 presents the semiquantitative scores assigned to each stage of epithelialization. Preoperative wounds generally exhibited limited epithelialization (scores 0–3) with predominance of granulation tissue, whereas final follow-up assessments consistently achieved complete epithelialization (score 5), helped by the reconstructive procedures. Intermediate postoperative evaluations showed gradual increases in epithelial coverage, reflecting effective wound closure over time. These findings provide visual, semi-quantitative, stage-specific evidence of healing dynamics and highlight the timeline of epithelial advancement in each patient.

## 5. Discussion

The inclusion of ozone therapy in the management of skin wounds in animals has begun to increase as the benefits are more widely recognized. This aspect is encouraged by studies conducted in both human and veterinary medicine that suggest this is due to the disinfectant, bactericidal, and regenerative properties of ozone [14,15]. Ozone therapy may be a relatively low-cost adjunct to wound management and may contribute to antimicrobial stewardship by potentially reducing antibiotic use and fighting antimicrobial resistance; however, comparative cost analyses and controlled studies are needed to confirm these potential advantages. It may also help lower the protocol doses of certain medications, such as anti-inflammatory and analgesic drugs [16,17,18].

One of the most common complications that occurs following skin flaps or skin grafts is represented by necrosis as a result of insufficient local blood supply [19]. Post-graft necrosis, a common complication in reconstructive surgery, was not observed in these four cases; while ozone may have contributed to this outcome, the absence of a control group precludes attributing causation. Ozone therapy can be an appropriate option, with proven properties, to support local skin flaps. By increasing the glycolysis rate of red blood cells, ozone therapy stimulates 2,3-diphosphoglycerate, resulting in an increase in oxygen released to tissues [20]. It induces the production of prostacyclin, which has a vasodilating effect. Blood circulation and oxygenation of ischemic tissues have also been improved as a result of the application of ozone therapy [21]. Hydrogen peroxide formed in plasma quickly diffuses into cells, and its appearance in the cellular cytoplasm triggers numerous biological effects. Reactive oxygen species (ROS) act by local vasodilation and stimulation of endogenous growth factors [22]. Moreover, ozone indirectly activates the non-specific immune system, resulting in processes such as the activation of phagocytosis and the production of interferon [23]. These aspects are essential in the survival of skin flaps as well as skin grafts.

Surviving a skin graft requires healthy and evenly distributed granulation tissue over the wound surface [24]. Given the tendency of cats to form granulation tissue in smaller amounts, it is ideal to apply therapies that stimulate this process. Patients presented in the study developed granulation tissue that was considered healthy and suitable for reconstructive surgery following the proposed ozone therapy program, which is consistent with a previous case presentation [25]. A higher level of oxygen in the wound area also supports the use of ozone because it helps stimulate the formation of granulation tissue, which enhances the healing process [9]. Also, the application of ozone therapy to human patients with diabetic foot ulcers facilitated their faster recovery compared to control groups [26].

Hydrogen peroxide increases the expression of vascular endothelial growth factor (VEGF) in human keratinocytes, an aspect that positively influences tissue revascularization [27]. In guinea pigs, topical application of ozonated olive oil can promote acute wound repair via increased expression of platelet-derived growth factor (PDGF), transforming growth factor beta (TGF-β), and VEGF [7]. While a direct effect in cats has not been confirmed, these findings provide plausible mechanisms for the clinical observations in our patients. One study evaluating the topical application of ozonized oil to skin flaps in rats noted a resurgence in VEGF levels [28]. Healing is noticeably faster by applying ozone therapy compared to conventional management methods, this being due to the positive impact of medical ozone on angiogenesis, fibroblast activity, and collagen production [6,29]. These aspects were clinically highlighted in a previous study [30]. Several explanations in this section are based on studies in other species, and their applicability to feline wound management requires cautious interpretation.

Wound infections prolong healing time due to the decreased activity of fibroblasts and collagenases produced by macrophages, granulocytes, and collagen-degrading bacteria [31]. In standard treatments, the use of antimicrobial therapy based on microbiological susceptibility examination is recommended if ozone, in certain methods of application, has a strong antibacterial activity. Topical ozone therapy enhances infected wound management, but it does not sufficiently penetrate deeper tissues or systemic circulation. Therefore, systemic antibiotics remain very important to eradicate bacteria in inaccessible compartments, making ozone therapy a valuable adjunct rather than a substitute. Clinical studies in human medicine have shown that combining ozone with standard antibacterial treatments improves healing rates, reduces inflammation and hospitalization, and may shorten antibiotic therapy duration. [11]. Similar synergistic effects have been reported in veterinary and experimental contexts, where ozone combined with antibiotics or disinfectants enhanced bacterial reduction and helped in the management of resistant organisms [32]. The oxidizing capacity of ozone appears to be the key element in carrying out antibacterial activity. Thus, ozone in contact with phospholipids in the bacterial membrane leads to its deterioration by oxidation [33]. These aspects have also been highlighted by electron microscopy [34]. Silva R.A. et al. in 2009 demonstrated the antibacterial capacity of ozone administered intraperitoneally in gaseous form in rats with induced peritonitis [35]. A single dose of 20 μg/mL ozone applied by nebulization in Petri dishes for 5 min under atmospheric pressure effectively inhibited the growth of all bacterial strains of *Escherichia coli*, *Staphylococcus aureus*, *Enterococcus faecalis*, *Acinetobacter baumannii*, and *Pseudomonas aeruginosa* [36]. These have pathogenic potential with known antimicrobial resistance. This antibacterial capacity of ozone was also clinically visible in patients presented in this study, benefiting from an average of 9 days of antimicrobial therapy. By directly exposing the wound to ozone by the bagging method and by lavages with ozonized saline solution, we can say that complementary ozone therapy through the two methods managed to maintain bacterial clearance of the wounds so that the healing process is not vitiated. Also, the ozonation of lavage liquids improves wound healing as well as cellular proliferation of keratinocytes in cell cultures [37,38,39]. According to the evolution of the 4th patient, who did not receive antimicrobial therapies at all, it may be possible that ozone therapy maintained the wound clearance. Overall, in our patients, no clinical signs of infection were observed following ozone therapy combined with the short course of systemic antibiotics, suggesting that this therapy may have contributed to maintaining a sterile wound environment; however, no post-treatment bacteriological cultures were performed, so antimicrobial efficacy was inferred from clinical aspects and evolution rather than confirmed microbiologically. It is a limitation of this case series that the synergistic effects of ozone therapy with antimicrobial treatment and surgical reconstruction cannot be precisely quantified. The favorable outcomes observed are likely the result of this multimodal approach.

The progressive decrease in ozone concentrations was due to its hormetic effect. Doses of 40–60 μg/mL aiming for antibacterial effect, and doses of 10–30 μg/mL for their regenerative effect [40].

Even if some of the mechanisms of action of ozone are not precisely, scientifically substantiated, being incompletely known, as for some practitioners, the approach of this complementary therapy may raise signs of uncertainty, some studies in the literature reinforce aspects of the safety of ozone administration in animals. It should be noted that despite the benefits observed, sometimes topical and subcutaneous administration of ozone, while explored for therapeutic purposes, could be associated with some safety concerns. Reported risks include inhalation toxicity, local tissue irritation, oxidative injuries, hypersensitivity reactions, and, in extremely rare instances, gas embolism when applied incorrectly. These potential adverse outcomes are further compounded by the lack of clearly worldwide accepted standardized treatment protocols and sometimes by the inconsistent quality of the equipment used, all of which in some cases raise uncertainty regarding their best application in medicine [14,32].

This case series represents a preliminary report, intending to initiate a new complementary therapeutic approach to prepare the tissues for reconstructive surgery and to maintain the microbial clearance after it in cats with extensive skin defects.

The limitations of this study consist of the heterogeneity of the wounds, the differences between individuals, the stage and aspect of the lesions when presented to our clinic, the reduced number of cases presented, the absence of post-treatment bacteriological cultures, and the absence of a control group. A higher number of individuals, histological evaluations, multiple staged bacteriological cultures, and a control group should be considered for a future case series on this topic.

## 6. Conclusions

The positive wound healing outcomes that may have resulted from the synergistic treatments of ozone therapy and reconstructive surgery, and given the wide safety margin of appropriately dosed ozone, suggest ozone therapy should be considered as a potentially useful complementary tool when treating extensive feline skin wounds. Within the limitations of this small, uncontrolled case series, preoperative and postoperative ozone therapy appeared to be well tolerated and may have supported wound bed preparation and postoperative healing in cats undergoing reconstructive surgery. These observations should be interpreted with caution, as definitive conclusions regarding efficacy cannot be drawn without a controlled, comparative study design. Nonetheless, ozone therapy in this context may merit further investigation as an accessible complementary treatment option.

## Figures and Tables

**Table 1:** Summary of cases, treatments, and outcomes.

Case	Etiology	Affected Area	Bacteria Involved	Wound Area	Method of Ozonetherapy Before Recontructive Surgery	Days of Persecundam Management	Type of Recontructive Surgery	Method of Ozonetherapy After Recontructive Surgery	Total Time to Healing (Days)
1	Traumatic origin	-left hind limb	*Staphylococcus* spp.	96.7 cm^2^	- bagging conc. 20–60 μg/mL for 5–20 min - perilesional infiltration with 15 μg/mL - lavage with ozonized saline solution at 60 μg/mL	15	Superficial caudal epigastric artery	Bagging	60
2	Post-amputation dehiscent wound	-left flank -sacral region, -groin -right hind limb	*Pseudomonas* spp.	163.2 cm^2^	- bagging conc. 20–60 μg/mL for 5–20 min - perilesional infiltration with 15 μg/mL - lavage with ozonized saline solution at 60 μg/mL	20	Local advancing skin flaps	Bagging	90
3	Unknow	-lumbosacral area	*Escherichia coli*, *Proteus* spp., and *Pseudomonas aeruginosa*	64.8 cm^2^	- bagging conc. 20–60 μg/mL for 5–20 min - perilesional infiltration with 15 μg/mL - lavage with ozonized saline solution at 60 μg/mL	31	Local advancing skin flaps and Free skin graft	Bagging	90
4	Car accident	-right forelimb’s dorsal metacarpal-phalangeal	Negative	34 cm^2^	- bagging conc. 20–60 μg/mL for 5–20 min - lavage with ozonized saline solution at 60 μg/mL	20	Free skin graft	Bagging	35

**Table 2:** Epithelialization progression scores at different healing stages in the treated patients.

Patient	Stage	Timepoint	Score (0–5)	Approx. Coverage (%)	Wound Clinical State
1	Initial presentation	day 0	0	0%	Large open wound with mostly necrotic superficial tissue, no epithelial margin.
	Mid-ozone therapy	day 15	1	≈15%	Visible epithelial migration from edges; central wound still granulating.
	Postoperative	day 29	4	≈95%	Near-complete coverage, small peripheral granulating areas.
	Final follow-up	day 60	5	100%	Complete epithelial coverage with hair regrowth.
2	Initial presentation	day 0	0	0%	Large open wound with isles of necrotic tissue, discrete epithelial margin and granulation.
	Mid-ozone therapy	day 20	2	≈40%	Well-developed granulation tissue with early epithelial advance from periphery.
	Postoperative	day 50	4	≈90%	Majority of surface covered with reduced flap retraction areas remain.
	Final follow-up	day 90	5	100%	Fully epithelialized, mature tissue, visible hair regrowth.
3	Initial presentation	day 0	0	0%	Open wound with isles of necrotic tissue, discrete granulation.
	Mid-ozone therapy	day 30	2	≈45%	Epithelial migration from margins more evident, granulation still prominent.
	Post 1st graft	day 33	3	≈65%	Epithelial migration from margins evident, granulation still prominent
	Post 2nd graft	day 65	4	≈95%	Almost complete epithelialization; few epithelializing isles remain
	Final follow-up	day 90	5	100%	Fully epithelialized, mature tissue with hair regrowth
4	Initial presentation	day 0	0	0%	Extensive open wound with necrotic margins, no epithelial tissue.
	Mid-ozone therapy	day 20	1	≈25%	Early epithelial growth at wound margins, predominantly granulation.
	Post-graft	day 29	4	≈90%	Majority of wound epithelialized; small residual granulating areas.
	Final follow-up	day 60	5	100%	Full epithelial coverage, hair regrowth present.

## Data Availability

The original contributions presented in this study are included in the article. Further inquiries can be directed to the corresponding author.
